# Global transgenerational gene expression dynamics in two newly synthesized allohexaploid wheat (*Triticum aestivum*) lines

**DOI:** 10.1186/1741-7007-10-3

**Published:** 2012-01-26

**Authors:** Bao Qi, Wei Huang, Bo Zhu, Xiaofang Zhong, Jianhua Guo, Na Zhao, Chunming Xu, Huakun Zhang, Jinsong Pang, Fangpu Han, Bao Liu

**Affiliations:** 1 Key Laboratory of Molecular Epigenetics of MOE and Institute of Genetics and Cytology, Northeast Normal University, Changchun 130024, China; 2 Key Laboratory of Applied Statistics of MOE, Northeast Normal University, Changchun 130024, China; 3 Institute of Genetics and Developmental Biology, Chinese Academy of Sciences, Beijing 101110, China; 4 Department of Agronomy, Jilin Agricultural University, Changchun 130118, China

## Abstract

**Background:**

Alteration in gene expression resulting from allopolyploidization is a prominent feature in plants, but its spectrum and extent are not fully known. Common wheat (*Triticum aestivum*) was formed via allohexaploidization about 10,000 years ago, and became the most important crop plant. To gain further insights into the genome-wide transcriptional dynamics associated with the onset of common wheat formation, we conducted microarray-based genome-wide gene expression analysis on two newly synthesized allohexaploid wheat lines with chromosomal stability and a genome constitution analogous to that of the present-day common wheat.

**Results:**

Multi-color GISH (genomic *in situ *hybridization) was used to identify individual plants from two nascent allohexaploid wheat lines between *Triticum turgidum *(2n = 4x = 28; genome BBAA) and *Aegilops tauschii *(2n = 2x = 14; genome DD), which had a stable chromosomal constitution analogous to that of common wheat (2n = 6x = 42; genome BBAADD). Genome-wide analysis of gene expression was performed for these allohexaploid lines along with their parental plants from *T. turgidum *and *Ae. tauschii*, using the Affymetrix Gene Chip Wheat Genome-Array. Comparison with the parental plants coupled with inclusion of empirical mid-parent values (MPVs) revealed that whereas the great majority of genes showed the expected parental additivity, two major patterns of alteration in gene expression in the allohexaploid lines were identified: parental dominance expression and non-additive expression. Genes involved in each of the two altered expression patterns could be classified into three distinct groups, stochastic, heritable and persistent, based on their transgenerational heritability and inter-line conservation. Strikingly, whereas both altered patterns of gene expression showed a propensity of inheritance, identity of the involved genes was highly stochastic, consistent with the involvement of diverse Gene Ontology (GO) terms. Nonetheless, those genes showing non-additive expression exhibited a significant enrichment for vesicle-function.

**Conclusions:**

Our results show that two patterns of global alteration in gene expression are conditioned by allohexaploidization in wheat, that is, parental dominance expression and non-additive expression. Both altered patterns of gene expression but not the identity of the genes involved are likely to play functional roles in stabilization and establishment of the newly formed allohexaploid plants, and hence, relevant to speciation and evolution of *T. aestivum*.

## Background

The widespread existence of allopolyploidy in the plant kingdom points to its important role in the evolution of many groups of plants [1-5]. Yet, reuniting divergent genomes from different species in one nucleus likely represents a traumatic experience that the newly formed allopolyploids must go through to survive and adapt. Conceivably, substantial reconciliation of incompatibility is required at the very early stages following allopolyploidization to enable the newly formed individuals to recover from the "genome shock" [6] and establish as new species.

We are still largely ignorant about the means and their underlying mechanisms whereby compatibility is accomplished at the onset of allopolyploidy. However, recent studies conducted over the last decade in diverse plant taxa have provided novel insights into the seemingly intangible enigma; the most striking being the documentation that allopolyploidization triggers instantaneous genetic and epigenetic changes that enable altered trajectories of gene regulation [7-14]. These rapid and non-Mendelian genetic, epigenetic and regulatory changes are thought as important in ameliorating the hurdles related to immediate accommodation of nascent allopolyploids, and may contribute to their establishment and evolution as competitive new species [1,4,15-25]. Paradoxically, initial genomic instability was not detected in all studied cases of successful speciation via allopolyploidy [26-28]. It is, however, important to note that alteration in gene expression appears to represent a consensus feature of nascent plant allopolyploidy involving diverse taxa [21,29-32]. Moreover, the immediate alteration in gene expression may provide the basis for the evolution of homoeologue-specific tuning or partitioning -- a unique property of allopolyploid species [4,20,33-36]. Maintenance of homoeologue-specific tuning or partitioning in an allopolyploid species requires cytological diploidization, and, hence, disomic inheritance, which conceivably entails timely functional differentiation among homeologous chromosomes [13].

Common wheat (*Triticum aestivum *L.) originated only about 10,000 years ago [37] from hybridization event(s) -- most probably between a domesticated form of tetraploid wheat, *T. turgidum *(for example, ssp. *durum *or the more primitive ssp. *parvicoccum*, genome BBAA) with a diploid goat grass species, *Aegilops tauschii *(genome DD) [37-41]. Common wheat thus provides a classic example of formation of a new species in a single step. Deciphering the process and understanding its mechanistic underpinning is of great evolutionary interest. Exploring the pattern and spectrum of global gene expression changes associated with nascent wheat allohexaploidization represents an essential step towards this goal.

Hitherto, there are three reports on global gene expression changes associated with nascent allopolyploidization in wheat, all employing genetically stable synthetic allohexaploid lines [42-44]. In the study by Pumphrey *et al*.[42], it was found that as high as 16% of the 825 analyzed genes selected from a 70-mer customer wheat oligonucleotide microarray displayed non-additive expression in a first generation synthetic allohexaploid line. In contrast, the study by Chagué *et al*. [44] documented that the great majority (*ca*. 93%) of 30,000 transcripts analyzed showed additive expression and, hence, leaving only about 7% of genes as non-additively expressed in two synthetic lines. The expression patterns were highly stable across two generations and fairly consistent among the two synthetic lines, which shared the same tetraploid parental genotype [44]. The third study focused only on transcripts that have detectable parent-specific features (PSF), and 19% of these genes showed non-additive expression [43]. The striking discrepancy in the proportions of non-additively expressed genes associated with nascent allohexaploidization for the same species (common wheat) by the three studies could be due to various factors, such as differences in the developmental stage of the leaf tissue investigated or the different organs studied (leaf vs. shoot). Most likely, the differences are due to the parental genotypes used [28,44]. Apparently, further independent investigations are needed to determine the molecular, cell biological and physiological opportunities provided by genome-wide combinations in novel syntheses of allohexaploid wheat.

The present study was aimed to address: (1) pattern and spectrum of changes in global gene expression at early generations of nascent allohexaploid wheat lines with chromosomal stability; (2) characteristics of the genes showing altered expression patterns, and their possible functional relevance; and (3) transgenerational heritability and inter-line conservation of the novel expression patterns.

## Results

### Cytological characterization of the two sets of newly formed allohexaploid wheat lines

**Figure 1:**
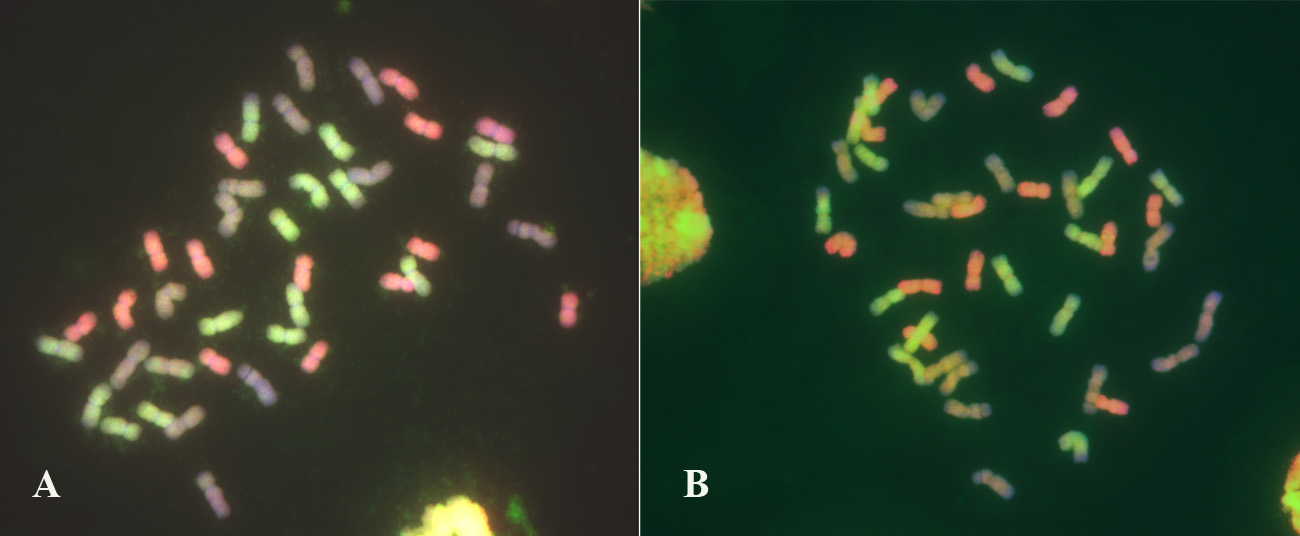
**Genomic integrity of the two newly formed allohexaploid wheat lines**. Multi-color GISH analysis of root-tip cells of the two newly formed allohexaploid lines, Allo-AT5 **(A) **and Allo-AT9 **(B)**, reveal genome integrity at the chromosomal level. The green-, pink-, and brown-coloration chromosomes are of the AA, BB and DD genomes, respectively.

We meticulously determined the chromosome configurations on root-tip mitosis of each individual plant for both nascent allohexaploid lines by the multicolor GISH technique [45]. We found that chromosomal numerical and structural variations, including aneuploidy and/or inter-genomic translocations, occurred in certain proportions of the plants in each line at both studied generations, S4 and S5 (data to be published elsewhere). Nonetheless, the two lines are considered as fairly stable at the chromosomal level compared with some other nascent allopolyploid plants (for example, *Brassica*), which is consistent with their tetraploid parental genotypes harboring the *Ph1 *gene that suppresses meiotic pairing between homeologous chromosomes and, hence, ensuring diploid-like meiosis [28,37,46]. Inclusion of the tetraploid parents containing the functional *Ph1 *gene in the synthesis of the allohexaploid wheat lines is important for evolutionary relevance as this is the case in the formation of natural common wheat [37]. For this study, only those individual plants that showed complete transgenerational chromosomal integrity as judged by the multi-color GISH were used as the RNA sources. These plants had a genome constitution consisting of three intact genomes corresponding to the B, A and D genomes of common wheat (Figure 1).

### Gene expression divergence between the parental plants of *T. turgidum *and *Ae. tauschii*, and conservation between the two subspecies of *T. turgidum*

The Affymetrix GeneChip^® ^Wheat Genome Array (The Affymetrix, INC.Santa Clara, CA, USA) contained 61,129 probe-sets representing 55,052 different genes that mapped across the common wheat genome. These genes are certainly not all expressed at a particular developmental stage of a given tissue, and we detected 29,650 genes that showed reliable expression (based on MAS5 flags analysis) between the two biological replications in the second-seedling-leaf tissue at the three-leaf-stage.

**Figure 2:**
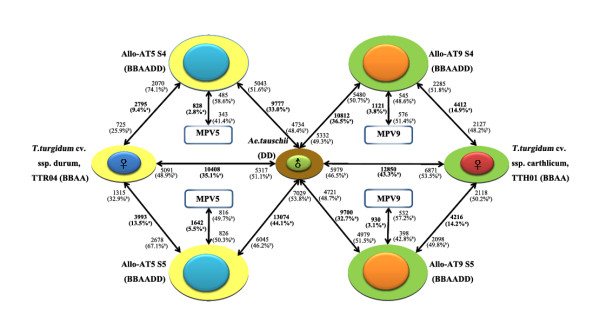
**Summary of microarray-based differential gene expression between each allohexaploid line and its corresponding parental species**. Diagrammatic illustration of parentage of and relationship between the two nascent allohexaploid wheat lines (Allo-AT5 and Allo-AT9) used in this study, and summary of the microarray-based genomewide difference in gene expression between each allohexaploid wheat line and its corresponding parental species, in two successive selfed generations (S4 and S5). The empirically measured mid-parent values (MPVs) for each allohexaploid line were included in all relevant comparisons. Bold text denotes the total number and fraction (%) of genes diagnosed as differentially expressed in each comparison, and the plain text indicates the number and fraction (%) of up-regulated genes of the total differentially expressed genes. For example, 10,408 genes are differentially expressed between the parental species, *T. turgidum*, ssp. *durum *cv. TTR04 and *Ae. tauschii*, line TQ27, of which 5,091 and 5,317 genes were up-regulated in TTR04 and TQ27, respectively. ^a^of all the expressed genes; bof the total number of differentially expressed genes between each comparison.

We compared the whole transcriptomic difference between each pair of the parental species, *T. turgidum *(ssp. *durum*, cv. TTR04) vs. *Ae. tauschii *(line TQ27), and *T. turgidum *(ssp. *carthlicum*, cv. TTH01) vs. *Ae. tauschii *(line TQ27) (Figure 2 and Additional file 1). Because in both combinations, TQ27 served as the paternal parent, the transcriptome of TQ27 was used as a reference. Compared with TQ27, 5,091 (48.9%) and 6,871 (53.5%) genes were up-regulated (*P *value < 0.05) in TTR04 and TTH01, respectively; whereas 5,317 (51.1%) and 5,979 (46.5%) genes were down-regulated in the two tetraploid wheat lines (Figure 2 and Additional file 1). Taken together, the percentages for the transcriptomic divergence between each pair of the parental species were calculated as 35.1% (TTR04 vs. TQ27) and 43.3% (TTH01 vs. TQ27) of all expressed genes, respectively (Figure 2 and Additional file 1). As expected, a large number and proportion (8,727, 60.1%) of all the differentially expressed genes (14,531) between a given tetraploid and the diploid parental species were overlapping (see Additional file 2), and accordingly, the number of genes showing inter-subspecific difference between *durum *and *carthlicum *was relatively small (2,459, 8.3%) (see Additional file 1).

### Differential parental contribution and parental dominance gene expression in the two nascent allohexaploid wheat lines

We compared the expression difference of each of the two synthetic allohexaploid wheat lines across two selfed generations directly with their tetraploid and diploid parental species. The number of differentially expressed genes between Allo-AT5S4 and its tetraploid parent *T. turgidum *ssp. *durum *amounted to only 9.4% and to its diploid parent *Ae. tauschii *to 33.0%. In the case of the Allo-AT9S4 the frequency of differentially expressed genes between the allohexaploid and its tetraploid *T. turgidum *ssp. *carthlicum *was 14.9% and with its diploid parent 36.5% (Figure 2 and Additional File 3). This indicates that the overall transcriptome of the nascent allohexaploid lines was more similar to that of *T. turgidum *than to *Ae. tauschii*, consistent with the two-third and one-third genomic contributions by the former and the later, respectively. Notably, the between-generation difference with respect to the number of differentially expressed genes for a given allohexaploid line vs. its parental species was evident (Figure 2). Notably, the two allohexaploid lines showed contrasting trends in the number of genes showing differential expression from their parental species across the two successive generations, S4 and S5 (Figure 2), underscoring genetic context-dependent differential expression dynamics in the two nascent allohexaploid lines.

For those genes that were expressed differentially between the parental species, their expression levels in the allohexaploid lines could be statistically equal to one of the parents but different from the other; that is, they display expression bias towards one of the two parents. This phenomenon has been systematically investigated in allotetraploid cotton, and was termed "parental dominance gene expression" [31,36].

**Figure 3:**
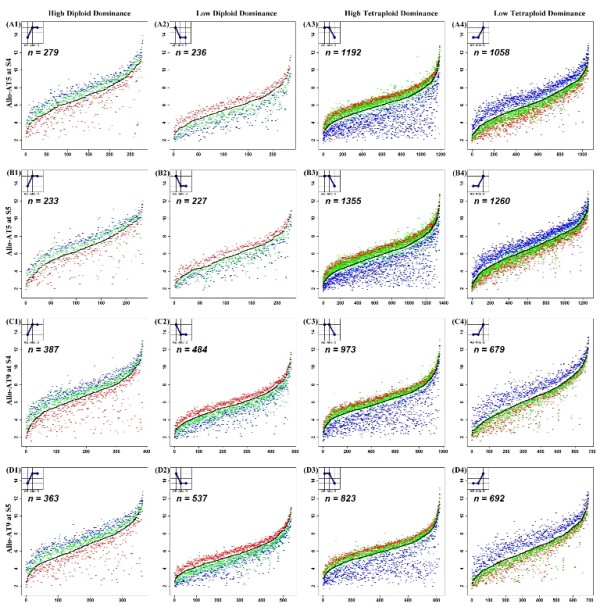
**Differential parental contribution and expression dominance in each allohexaploid line**. Comparison of gene expression levels among the nascent allohexaploid wheat line (Allo-AT5 or Allo-AT9), their mid-parent values (MPVs) and those of their parental species (see Figure 2 for details). Genes were ordered on X-axis by their normalized expression levels in MPVs, and the gene expression level was presented as log-ratio of normalized microarray data. Red-TTR04 or TTH01, blue-TQ27, green--Allo-AT5 or Allo-AT9 at the S4 or S5 generation, the black curve--MPVs. A1-D1, expression levels of high diploid dominance expression genes in each allohexaploid line; A2-D2, expression levels of low diploid dominance expression genes; A3-D3, expression levels of high tetraploid dominance expression genes; A4-D4, expression levels of low tetraploid dominance expression genes. The inserted graphs illustrate the expression patterns among the tetraploid, diploid and the allohexaploid, wherein statistically differential expression is denoted by different vertical levels.

Since the parental species were of different ploidy-level, a conceivable contributing factor to the seemingly parental biased expression in the allohexaploid lines was differential hybridization affinity by the two parental species' cDNAs on the same array [44]. We used the experimentally measured mid-parent values (MPVs) for each parental species combination which was generated by mixing the RNAs of parental lines (*T. turgidum *and *Ae. tauschii*) at a ratio of 2:1 to exclude this confounding factor. The rationale was: those genes that appeared as biased expression in synthetic allopolyploids but actually were due to differential hybridization-affinity would also show "biased expression" in MPVs, and were excluded (Figure 3 and Additional file 4). Thus, we confined our analysis to those genes that exhibited parental biased expression in the allohexaploid lines but not in the corresponding MPVs, that is, the *bona fide *parental expression dominance genes resulting from allopolyploidy-specific tuning.

Broadly, two categories of parental expression dominance were recognized, that is, diploid parental dominance and tetraploid parental dominance, each category including two patterns. For diploid parental dominance: pattern one was that expression levels in the diploid were greater than the tetraploid, and the allohexaploid values largely fell above the MPV, which were statistically equal to the higher expression levels of the diploid, but different from the lower expression levels of the tetraploid (Figure 3A1, B1, C1, and 3D1); pattern two was that expression levels of the diploid were lower than the tetraploid, and the allohexaploid values largely fell below the MPV, which were statistically equal to the lower expression levels of the diploid but different from the higher expression levels of the tetraploid (Figure 3A2, B2, C2, and 3D2). For tetraploid parental dominance: pattern one was that expression levels in the tetraploid were greater than the diploid, and the allohexaploid values largely fell above the MPV, which were statistically equal to the higher expression levels of the tetraploid, but different from the lower expression levels of the diploid (Figure 3A3, B3, C3 and 3D3); pattern two was that expression levels of the tetraploid were lower than the diploid, and the allohexaploid values largely fell below the MPV, which were statistically equal to the lower expression levels of the tetraploid but different from the higher expression levels of the diploid (Figures 3A4, B4, C4, and 3D4).

**Figure 4:**
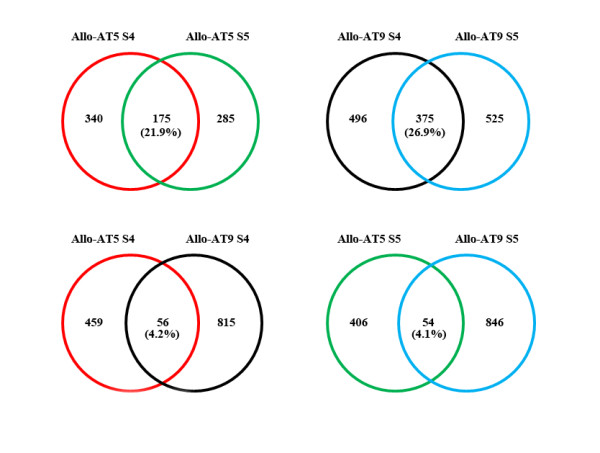
**Conservation of diploid parental expression dominance genes within and between the allohexaploid lines**. Venn diagrams illustrating the number and proportion (%) of those diploid parent-dominance expression genes that are common between the two selfed generations (S4 and S5) for a given nascent allohexaploid line (Allo-AT5 or Allo-AT9), and those that are common between the two lines (Allo-AT5 and Allo-AT9) at a given generation (S4 or S5).

In general, the number of genes showing diploid parental dominance was much fewer than those showing tetraploid parental dominance in each allohexaploid line at each generation (see Additional file 4). The parental dominance expression genes in the allohexaploid lines showed either statistically additive or non-additive expression levels, with the former being the great majority while the later accounting for less than 21.5% of these genes (data not shown). This accorded well with the earlier results in both cotton [31,36] and wheat [44]. It should be emphasized that this "phenotypic additivity" in expression pattern shown by the parental dominance expression genes may not solely result from the summing up of parental transcripts in which the occurrence of expression alterations did not transgress the expected parental additive expression range; rather, they could be caused by marked, yet compensatory, changes between the homeologous alleles.

**Figure 5:**
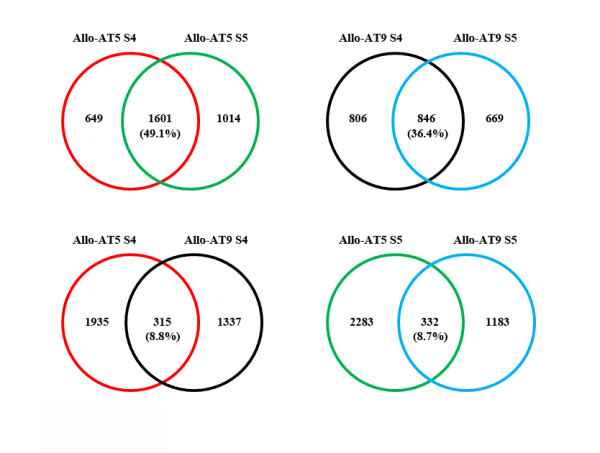
**Conservation of tetraploid parental expression dominance genes within and between the allohexaploid lines**. Venn diagrams illustrating the number and proportion (%) of those tetraploid parent-dominance expression genes that are common between the two selfed generations (S4 and S5) for a given nascent allohexaploid line (Allo-AT5 or Allo-AT9), and those that are common between the two nascent allohexaploid lines (Allo-AT5 and Allo-AT9) at a given generation (S4 or S5).

We noted that the parental dominance expression genes showed low degrees of conservation. That is, the proportions of these genes that were overlapping between the two generations of a given allohexaploid line and between the two lines at a given generation were small. Specifically, there were only 175 and 375 diploid parental dominance expression genes that were overlapped between the S4 and S5 generations in Allo-AT5 and Allo-AT9, respectively, which accounted for 21.9% and 26.9% of the total diploid parental dominance expression genes in the two lines (Figure 4). Similarly, there were 1,601 and 846 tetraploid parental dominance expression genes that were overlapped between the S4 and S5 generations in Allo-AT5 and Allo-AT9 and which accounted for 49.1% and 36.4% of the total tetraploid parental expression dominance genes in the two allohexaploid lines, respectively (Figure 5). More limited conservation of dominance expression genes was observed between the two allohexaploid lines at a given generation, which applied particularly so for those of the diploid parent. Specifically, there were 4.2% (56/1,330) and 4.1% (54/1,306) diploid parental dominance expression genes being shared by the two lines at S4 and S5, respectively, whereas the proportions were 8.8% (315/3,587) and 8.7% (332/3,798) for the tetraploid parental dominance expression genes at the two generations (Figures 4 and 5). Expectedly, there were only 14 and 133 genes with expression patterns that were consistently biased toward the diploid and tetraploid parental species, respectively, in both lines at both generations (see Additional file 5).

The foregoing results indicated that the parental dominance expression genes in the allohexaploid wheat lines could be classified into three distinct groups, "stochastic" (group I) referring to those that were specific to one line (Allo-AT5 or Allo-AT9) at one generation (S4 or S5) only, "heritable" (group II) referring to those that were specific to one line at both generations, and "persistent" (group III) referring to those that were commonly detected in both lines at both generations. Consistent with the above analysis, the group II and III genes were of markedly greater proportions among the total tetraploid parental dominance expression genes than those of the diploid (Figures 4 and 5), suggesting that allopolyploidy-regulated gene expression is more stochastic in the transcriptome contributed by the diploid parent than in that by the tetraploid parent.

### Non-additive gene expression in the nascent allohexaploid wheat lines

Another prominent pattern of altered gene expression commonly associated with nascent plant allopolyploidy is non-additivity. By definition, if the expression level of a gene in an allopolyploid did not change from that of their parental lines, then its expression level would be statistically equal to that of the experimentally measured mid-parent values (MPVs), described above; otherwise, it is defined as non-additive. At this point, it appeared important to distinguish between the two expression patterns. While the "parental dominance gene expression" refers to the statistically expression similarity in an allopolyploid to one of the parents but different from the other, the "non-additivity" refers to those genes the expression levels of which deviated from the empirically measured mid-parent values (MPVs). Genes of these two expressing patterns were neither mutually exclusive nor inclusive. For example, a gene may show expression deviation from that of the MPVs, that is, non-additivity, but at the same time its expression level may be statistically equal to one of the parents but different from the other, that is, expression dominance. Conversely, a gene could have shown parental dominance expression, but statistically did not deviate from the MPVs, and hence, was categorized as additive expression.

**Figure 6:**
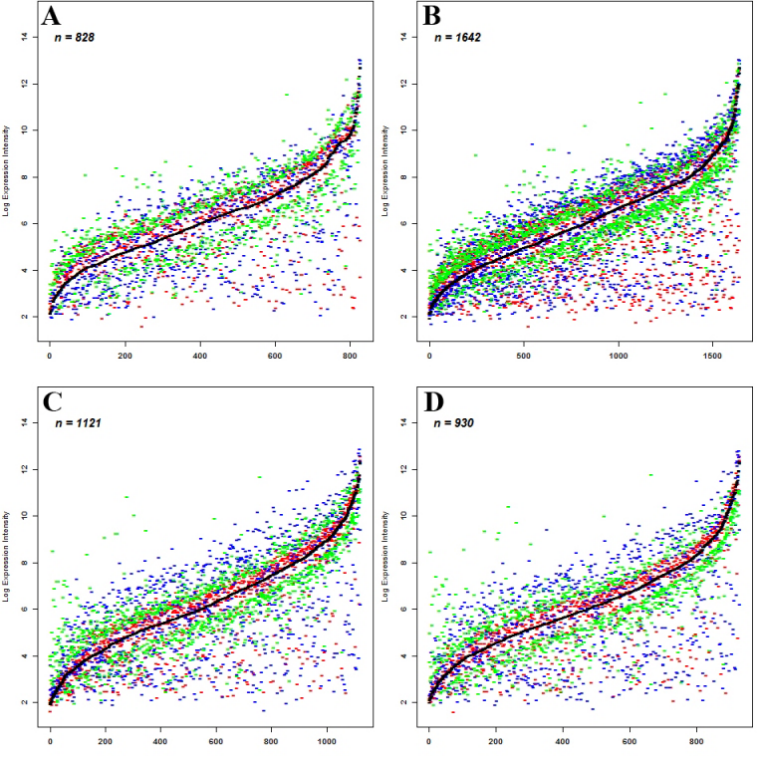
**Non-additive expression in each of the two allohexaploid lines across two generations**. Comparison of gene expression levels (probe hybridization intensity values of the microarray data), presented as log-ratio of normalized data, obtained for each transcript of the non-additive expressing genes, among the nascent allohexaploid line (Allo-AT5 or Allo-AT9), at two successive selfed generations (S4 and S5), their mid-parent values (MPVs) and those of their parental species (see Figure 2 for details). Genes were ordered by their normalized expression levels in MPVs (black curve), Red-TTR04 or TTH01, blue-TQ27, green--Allo-AT5 or Allo-AT9 at the S4 or S5 generation. **(A) **Non-additive expression genes in Allo-AT5 at S4; **(B) **non-additive expression genes in Allo-AT5 at S5; **(C) **non-additive expression genes in Allo-AT9 at S4; and **(D) **non-additive expression genes in Allo-AT9 at S5.

We quantitatively investigated the phenomenon of non-additivity by comparing the microarray-based gene expression levels of the two nascent allohexaploid wheat lines at two successive generations against the empirically measured MPVs (see Methods). We found that a great majority of the expressed genes showed additive expression compared with the corresponding MPVs, which if averaged across the two generations (S4 and S5), accounted for 95.8% and 96.5% of the total expressed genes in Allo-AT5 and Allo-AT9, respectively (Table 1 and Figure 2). Nonetheless, deviation from additivity in gene expression occurred in both lines, though with marked difference in regard to its expression pattern and preponderance of transgenerational inheritance (Table 1 and Figure 6). For example, Allo-AT5 showed more inter-generational difference (2.8% at S4 vs. 5.5% at S5) than Allo-AT9 (3.8% at S4 vs. 3.1% at S5) (Table 1). Also, higher proportions showed up- rather than down-regulation in Allo-AT5 (average 53.1% vs. 46.9%), but the opposite was true in Allo-AT9 (average 46.0% vs. 54.0%) (Table 1). A consensus feature for the non-additive genes across lines and generations was their over-representation by those showing parental differential or divergent expression (Table 1), as also documented previously in various plant taxa [42-44,47-50].

**Table 1:** Pattern and extent of transgenerational, non-additive gene expression in each of the synthetic allohexaploid lines

Synthetic line and generation	Allo-AT5			Allo-AT9		
	
	S4	S5	**Aver**.	S4	S5	**Aver**.
Total no. and (%^a^) of nonadditively expressed genes	828 (2.8)	1,642 (5.5)	1,235 (4.2)	1,121 (3.8)	930 (3.1)	1,026 (3.5)

No. and (%^b^) of up-regulated genes	485 (58.6)	826 (50.3)	656 (53.1)	545 (48.6)	398 (42.8)	472 (46.0)

No. and (%^b^) of down-regulated genes	343 (41.4)	816 (49.7)	580 (46.9)	576 (51.4)	532 (57.2)	554 (54.0)

No. and (%^b^) of the total as well as up- and down-regulated non-additively expressed genes that matched the genes showing differential expression between parental species	T-483 (58.3) Up- 237 (28.6) Down: 246 (29.7)	T-1055 (64.3) Up- 426 (25.9) Down: 629 (38.3)	T-769 (62.3) Up- 332 (26.8) Down: 438 (35.4)	T-741 (66.1) Up- 316 (28.2) Down: 425 (37.9)	T-613 (65.9) Up- 203 (21.8) Down: 410 (44.1)	T-677 (66.0) Up- 260 (25.3) Down: 418 (40.7)

No. and (%^b^) of high-diploid Parental dominance genes	31 (3.7)	41 (2.5)	36 (2.9)	97 (8.7)	79 (8.5)	88 (8.6)

No. and (%^b^) of high-tetraploid Parental dominance genes	178 (21.5)	342 (20.8)	260 (21.1)	112 (10.0)	64 (6.9)	88 (8.6)

No. and (%^b^) of Low-diploid Parental dominance genes	64 (7.7)	62 (3.8)	63 (5.1)	110 (9.8)	91 (9.8)	101 (9.8)

No. and (%^b^) of low-tetraploid parental dominance genes	78 (9.4)	190 (11.6)	134 (10.9)	129 (11.5)	115 (12.4)	122 (11.9)

No. and (%^b^) of over-dominance genes	44 (5.3)	94 (5.7)	69 (5.6)	108 (9.6)	65 (7.0)	87 (8.4)

No. and (%^b^) of under- Dominance genes	14 (1.7)	31 (1.9)	23 (1.8)	73 (6.5)	35 (3.8)	54 (5.3)

^a^of all expressed genes.

^b^of non-additively expressed genes.

The up- or down-regulated numbers of the nonadditively expressed genes.

The nonadditively expressed genes can be divided into six categories based on their expression: F represented the synthetic allohexaploid lines; P, paternal lines; and M, maternal lines. High-paternal dominance, F = P, F > M, P > M; high-maternal dominance, F = M, F > P, M > P; low-paternal dominance, F = P, F < M, P < M; low-maternal dominance, F = M, F < P, M < P; over-dominance, F > P and F > M; under-dominance, F < P and F < M.

According to criteria specified previously [42,44,47], the non-additive expressing genes could also be further classified into several patterns, including high- and low-diploid parental dominance, high- and low-tetraploid parental dominance, over-dominance and under-dominance (Table 1; Figure 6). The two lines manifested marked differences in the proportions of the non-additive genes showing the various patterns of expression. For example, in Allo-AT5, more tetraploid than diploid parental dominance expression genes were detected (21.1% vs. 2.9% and 10.9% vs. 5.1% for high- and low-parental dominance, respectively); in Allo-AT9, the number showing tetraploid parental dominance was the same as or similar to that showing diploid parental dominance (8.6% vs. 8.6% and 11.9% vs. 9.8% for high- and low-parental dominance, respectively). The average numbers of non-additive genes showing over- and under-dominance in Allo-AT5 (69 and 23, respectively) were slightly lower than those in Allo-AT9 (87 and 54 respectively); however, in both lines significantly more over- than under-dominance genes were detected (Table 1).

**Figure 7:**
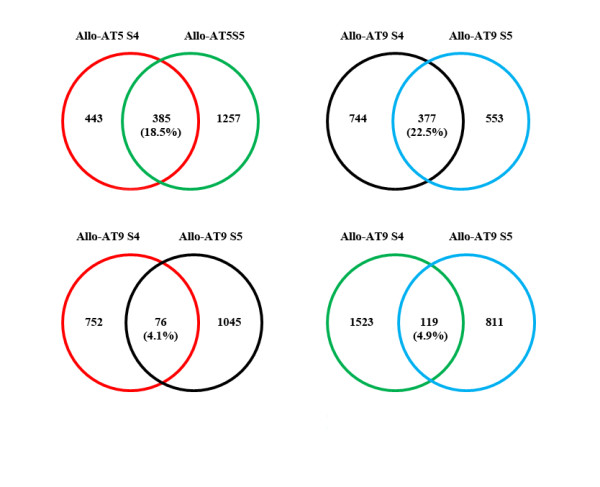
**Conservation of non-additive expression genes within and between the allohexaploid lines**. Venn diagrams illustrating the number and proportion (%) of those non-additive expression genes that are common between the two selfed generations (S4 and S5) for a given nascent allohexaploid line (Allo-AT5 or Allo-AT9), and those that are common between the two lines (Allo-AT5 and Allo-AT9) at a given generation (S4 or S5). Not shown are 27 genes that are common to both nascent allohexaploid lines (Allo-AT5 and Allo-AT9) at both generations (S4 and S5).

The proportions of common non-additive genes in the two successive generations of each line (termed "heritable non-additive genes") were low. Specifically, there were only 18.5% (385/2,085) and 22.5% (377/1,674) such genes being shared by the S4 and S5 generations in Allo-AT5 and Allo-AT9, respectively (Figure 7). This rendered the nonadditive genes that were specific to one allohexaploid line in only one generation (termed "stochastic nonadditive genes") a high proportion of the total non-additive genes, which comprised 81.5% and 77.5% in Allo-AT5 and Allo-AT9, respectively. If comparing the extent of overlaps of the nonadditive genes between the two allohexaploid lines at a given generation, the proportions were even lower, with only 4.1% (76/1,873) and 4.9% (119/2,453) at generations S4 and S5, respectively (Figure 7). Not shown in the figure was that there were only 27 non-additively expressed genes that were common to both lines at both generations, which were termed "persistent non-additive genes" (see Additional file 6).

### Validation of the microarray data by real-time quantitative (q)-RT-PCR in the nascent allohexaploid wheat lines

**Figure 8:**
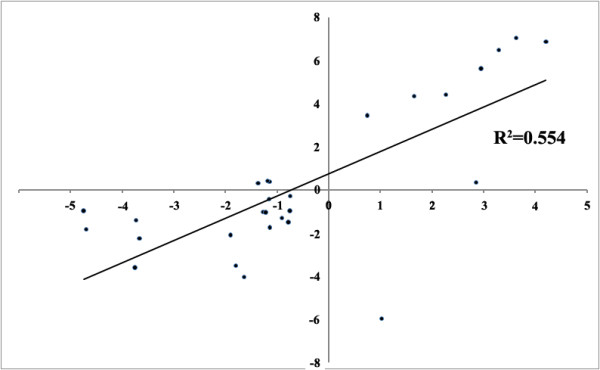
**Validation of the microarray data by (q) RT-PCR**. The ratio of non-additive gene expression (log2 scale) in the two nascent allohexaploid wheat lines was calculated according to the (q) RT-PCR experiment and plotted against the ratio calculated by microarray. The detailed information is given in Additional file 7, Table S6.

To verify the microarray data, we selected nine genes that showed non-additive expression in one or both of the two allohexaploid lines at one or both generations, and subjected the genes to (q)-RT-PCR analysis. These nine genes included the three groups of non-additively expressed genes, that is, stochastic, heritable and persistent and, therefore, 28 comparisons for non-additivity were generated across the two lines and the two generations (see Additional file 7). The expression level of each of these genes was compared with similarly constructed MPVs as used in the microarray experiments. Results indicated that of the 28 comparisons, 24 were confirmed as matching or highly similar to the microarray data for non-additive gene expression (Figure 8 and Additional file 7), thus confirming reliability of the microarray data and analysis.

### Enrichment for a specific category of genes involved in vesicle function by the non-additively expressed genes in the two nascent allohexaploid wheat lines

To investigate possible functional relevance of the two major groups of genes showing allohexaploid-specified regulation, that is, parental dominance and non-additivity, we conducted Gene Ontology (GO) analysis for each group, both as an entirety and as further divided subgroups.

**Figure 9:**
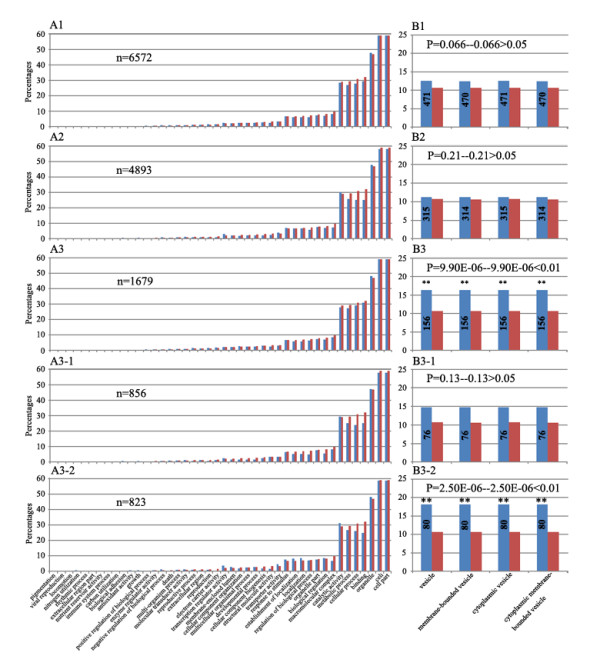
**Gene Ontology (GO) analysis of the parental expression dominance genes**. The *y*-axis is the percentages of genes mapped by the GO category terms: the percentages were calculated by the number of genes mapped to the particular GO category divided by the number of all genes. The *x*-axis is the GO category terms which were ordered by their relative abundance. The blue bars denote percentages for each category of all the annotated genes, and the red bars denote percentages of the GO categories of all the expressed genes detected in the studied leaf tissue (29,650 in total). The numbers inside the blue bars are the annotated genes in the specific GO categories. The enrichment FDRs (*P*-values) were given. ** Significant at the 0.01 statistic level. The GO analysis was performed both as an entirety and as distinct subgroups. **(A1) **All the parental expression dominance genes (6,572 in total) being considered as an entity; (A2) hose that showed additive expression (4,893 in total); (A3) those that showed non-additive expression (1,679 in total); (A3-1) those that showed non-additive expression in only one of the two generations (S4 or S5), that is, non-heritable (856 in total); (A3-2) those that showed non-additive expression in both generations (S4 and S5), that is, heritable (823 in total). **(B1) **The percentages of four GO annotation categories (all related to vesicle function) among all the parental expression dominance genes being considered as an entity; (B2) among those that showed additive expression; (B3) among those that showed non-additive expression; (B3-1) among those that showed non-additive expression in only one of the two generations (S4 or S5), that is, non-heritable; (B3-2) among those that showed non-additive expression in both generations (S4 and S5), that is, heritable.

**Figure 10:**
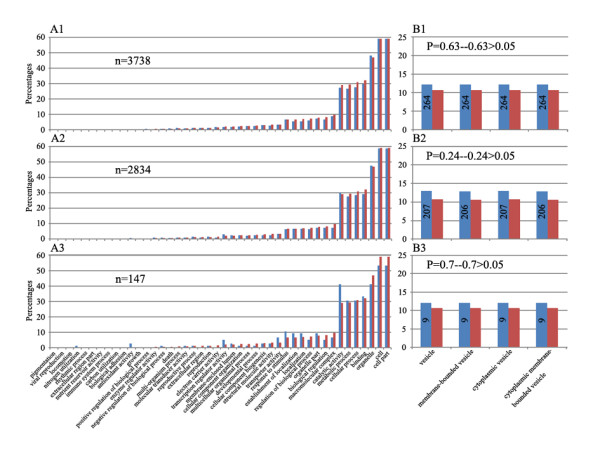
**Gene Ontology (GO) analysis of the parental expression dominance genes as three subgroups**. Meaning for the x- and y-axis and the bars are the same as in Figure 9. The GO analysis was performed for each of the three groups, I, II and III, classified based on inter-line conservation and transgenerational heritability (see main text for classification). All three groups pointed to the involvement of diverse functional pathways **(A)**, as in Figure 9, but according to this classification, no statistically significant enrichment for the vesicle function-related genes was found **(B)**.

First, for the parental dominance expression genes, we found that (1) diverse GO terms were involved, indicating that the genes showing this expression pattern had various molecular functions, participated in diverse biological processes, and localized to different cellular components (Figures 9A1-A3 and Figure 10A1-A3); (2) statistical tests for enrichment indicated that only those that exhibited the nonadditive expression pattern were enriched for a very specific GO category of the cellular component involved in vesicle function (Figures 9B1-B3 and Figure 10B1-B3), suggesting that this specific subgroup (showing expression nonadditivity) of the parental dominance genes is probably functionally required by the nascent allohexaploid wheat lines; (3) if these nonadditive expression genes were analyzed as transgenerationally heritable and non-heritable subgroups (Figure 9A3-1, -2), then only the heritable subgroup showed statistically significant enrichment for the vesicle-related genes (Figure 9B3-1, -2); (4) if the genes were analyzed as the three subgroups, stochastic, heritable and persistent (Figure 10), then none showed any enrichment (Figure 10B1-B3). Taken together, this analysis led to an important conclusion that only those parental dominance genes that are *non-additive as well as heritable *are enriched for the vesicle-related genes. This, however, becomes cryptic, if all the parental dominance genes were analyzed as a whole.

**Figure 11:**
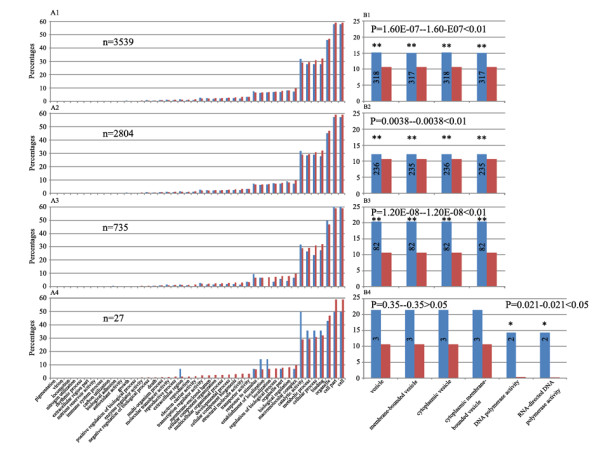
**Gene ontology (GO) analysis of non-additive expression genes**. Meaning for the x- and y-axis and the bars are the same as in Figure 9, the enrichment FDR was labeled on the top of the figures B1 to B4. * Significant level at FDR *P *< 0.05, ** significant level at FDR *P *< 0.01. The involvement of varies functional Gene Ontology categories of all the nonadditive expression genes (3,539 in total) in one or both of the nascent allohexaploid wheat lines (Allo-AT5 and Allo-AT9) **(A1)**; all the group I non-additive genes (2,804 in total) (A2); all the group II non-additive genes (735 in total) (A3); and all the group III non-additive genes (27 in total) (A4). The percentages of enriched GO functional categories of all the non-additive genes **(B1)**; the group I non-additive expression genes (B2); the group II genes (B3); and the group III genes (B4).

Second, we carried out GO analysis for the non-additively expressed genes, both as an entirety and as three distinct groups: stochastic (group I), heritable (group II), and consistent (group III). We found that: (1) similar to the situation of parental dominance expression genes, the non-additive genes as an entirety were also involved in diverse functional categories (Figure 11A1 to 11A4); (2) statistical analysis for enrichment indicated that, remarkably, the same GO terms of the cellular component, that is, those involved in vesicle function, showed significant enrichment, with those of group II manifested a higher degree of enrichment than group I (Figure 11B1 to 11B3); (3) the statistical insignificance of group III for those same GO-terms is apparently due to their too small number (27), and hence, lack of statistical power, rather than the absence of a real enrichment (Figure 11B4); and (4) interestingly, the group III genes also showed statistically meaningful homology to two DNA polymerases (Figure 11B4).

Taken the GO analysis results together for the two sets of genes specifically tuned by allopolyploidy, it is clear that only the non-additively expressed ones (irrespective of whether they showed parental dominance expression or not) manifested strong and specific enrichment for the small category of GO terms related to vesicle-function, with the subset of those exhibiting transgenerational inheritance being further enhanced for the enrichment.

## Discussion

In this study, we used the Affymetrix GeneChip^® ^Wheat Genome Array platform as was done in two of the three studies [43,44], as well as similar synthetic allohexaploid wheat lines as was used in one study [44]. Therefore, it is perhaps not surprising that our major results are more consistent with those of Chagué *et al*. [44] than to the other two studies. However, the synthetic lines we used also differ from those of Chagué *et al*. in three important aspects: (1) the two lines we used share the same genotype of the diploid goat-grass species, *Ae. tauschii*, as the paternal parent, but with different subspecies *durum *and *carthlicum *of *T. turgidum*, as the maternal parent, whereas the two lines used by Chagué *et al*. are with the same genotype of the tetraploid maternal parent but different genotypes of the diploid paternal parent; (2) our lines and those of Chagué *et al*. are of different generations following initial allopolyploidization, with ours being at the fourth and fifth selfed generations and those of Chagué *et al*. being at the immediate and first selfed generations (S0 and S1); (3) different tissues are used as the RNA source, that is, the second seedling-leaf (at the third-leaf stage) in our study and shoots (at the fifth-leaf stage) in the study of Chagué *et al*.. Therefore, being based on the same microarray platform and employing similar statistical methods, the results of our study have provided important comparisons as well as complementation to those of Chagué *et al*. [44]. For example, Chagué *et al*. [44] documented that the non-additively expressed genes between the S1 and S2 generations of one of the studied synthetic allohexaploid line were highly conserved. In contrast, we showed here that it was the *proportion *of the genes with the altered expression pattern that was conserved between the two successive generations (S5 and S6) of both allohexaploid lines studied, whereas *identity *of the genes *per se *were highly variable (detailed in following sections). These differences could be due to one or more of the different factors involved or the combinatory effects thereof. But it is clear, based on the results of Chagué *et al*. [44] and ours, that the early generations of allohexaploidy in wheat are extremely dynamic in gene expression, even in karyotypically normal and genetically stable plant individuals. We believe this double-edged property (genomic stability but transcriptomic dynamics) of nascent allopolyploidy is critical for bestowing stabilization on one hand and evolvability on the other of the newly formed allopolyploid plants towards establishment and speciation.

### Importance of chromosomal integrity in allohexaploid wheat evolution

It is well established by earlier cytological studies that, at the chromosomal level, the three constituent genomes of common wheat are largely intact. We, thus, analyzed the chromosomal constitution of each individual plant by the multicolor GISH technique [45], and chose only those plants with transgenerational chromosomal integrity for the microarray analysis. Therefore, our study has paid attention to this critical point that was unheeded by most previous investigations, except the one by Chagué *et al*.[44].

### Conservation in expression pattern but stochasticity in gene identity characterize both parental dominance expression genes and non-additive expression genes

A major pattern of altered gene expression in the nascent allohexaploid wheat lines is parental dominance expression. The experimental MPVs enabled unequivocal exclusion of those genes for which the detected biased expression was due to differential hybridization affinity by the parental species' cDNAs to the same array, and therefore, allowed analysis of the *bona fide *parental expression dominance genes resulting from allohexaploidy-specified tuning. Similarly, comparisons with the MPVs allowed unambiguous identification of the genes showing non-additive expression in the nascent allohexaploid lines. An important generalization that emerged from these analyses is that both the parental dominance expression genes and non-additive expression genes can be divided into three distinct groups depending on whether they occurred only in one allohexaploid line at only one generation, that is, stochastic (group I), in one line at both generations, that is, heritable (group II) or in both lines at both generations, that is, persistent (group III). Although this classification is meaningful only in a relative sense, its mere occurrence implicates an important attribute; that is, if the tuned expression is important for the newly formed allohexaploid lines, then it is likely *the pattern of expression *rather than *identity of the genes showing the pattern of expression *bears the relevance. This novel feature of allopolyploidy-specified gene regulation has not been reported previously, but it is reminiscent of the findings in allotetraploid cotton, in which the parental dominance expression was found to be even in opposite directions in different allotetraploid lines but with similar proportions of genes showing the pattern [31,36,51]. This feature of altered genomewide expression patterns also bears remarkable resemblance to the phenomenon of nucleoli dominance dictated by localized epigenetic difference, that is, stochastic silencing of one of the parental rRNA genes in an allopolyploid [21,52,53], thus further suggesting possible commonality of the phenomenon in nascent plant allopolyploids.

It is important to note that the majority of the genes, albeit showing parental dominance expression in the allohexaploid wheat lines, did not transgress the parental additive expression-range relative to the corresponding MPVs, as were found in cotton [31,36,49,50]. This is interesting given that the "appeared" expression additivity for this set of genes was actually achieved by allohexaploidy-specific tuning rather than merely the adding-up of parental transcripts, suggesting that a too high- or low-level of expression is likely detrimental and, hence, is being selected against.

### Non-additive gene expression in the newly formed allohexaploid wheat lines

Deviation from additivity in expression pattern of genes was commonly observed in previous studies, albeit the spectrum of deviation can be dramatically variable. Although the functional relevance of non-additive gene expression remains largely unexplored [54], it is suggested that the non-additivity-bestowed diverse novel gene expression patterns may provide for variations upon which selection can act [16,36,55,56].

It is conceivable that for non-additive gene expression to play a biologically meaningful role, transgenerational heritability of the altered expression patterns and/or the genes is required. Indeed, several studies have indicated that the non-additive expression patterns can be established at the initial stages of allopolyploidization but with protracted effects over evolutionary timescales [5,21,24,29,34,48,57]. Results of this study suggest that it is the altered pattern of non-additive gene expression rather than the involved genes *per se *that showed a propensity of transgenerational inheritance, as evidenced by the results that the proportions of genes showing the altered expression patterns were highly similar between the two successive generations but the identities of the genes are highly variable. This novel paradigm of allopolyploidy-associated gene expression has not been reported previously.

### Possible requirements for specific tuning of genes involved in vesicle function by the nascent allohexaploid wheat lines

Gene Ontology (GO) analysis indicated that both groups of genes showing allopolyploidy-specific tuning, that is, parental dominance expression and non-additive expression, are involved in diverse functional GO-categories. Nevertheless, detailed analysis revealed that only those genes that showed non-additive expression, regardless of whether they are exhibiting parental dominance expression or not, are enriched for a very specific GO-category involved in vesicle function. Moreover, the enrichment was further enhanced for a subset of these genes showing transgenerational heritability. This novel observation strongly implicates a functional relevance of non-additive gene expression being associated with nascent allopolyploidy, which makes sense given that an immediate outcome of allopolyploidization is enlarged cell volume but reduced surface-area to volume ratio [4,58], and, therefore, tuned non-additive expression of vesicle-related genes may help circumvent this dilemma.

The molecular basis underlying the allopolyploidy-regulated gene expression remains to be fully elucidated [20,21,34,55,59,60]. It has been proposed that both genetic regulatory mechanisms, such as *cis- *and *trans-*interactions [61], and epigenetic alterations, such as altered DNA methylation and small RNA biogenesis [62,63], likely play important roles. Further studies are needed to explore the exact roles played by these mechanisms in the allohexaploidization of wheat.

## Conclusions

This study showed that the early stages of allohexaploidization, leading to the formation of allohexaploid wheat, exhibited two major patterns of global gene expression alteration: parental dominance expression and non-additive expression. The mechanisms underpinning the expression changes associated with nascent allopolyploidy remain largely unknown [61,64], but chromosomal instability is not a causal factor. Instead, molecular interactions, including altered *cis-*/*trans-*regulation [61,63], dosage balance/compensation [16,56], gene elimination and/or epigenetic remodeling [17,63], brought about by the sudden merging of divergent parental species' genomes, are conceivably responsible [17]. Genes involved in each altered expression pattern could be classified into three distinct groups: stochastic, heritable and persistent, based on their transgenerational heritability and inter-line conservation. Importantly, whereas both altered patterns of gene expression showed a propensity of transgenerational inheritance, identity of the involved genes is highly stochastic. Accordingly, diverse Gene Ontology (GO) terms were implicated with both patterns of altered gene expression; however, those showing non-additive expression manifested a significant enrichment for a specific group of proteins associated with vesicle function. Our results suggest that global alteration in gene expression conditioned by nascent allopolyploidy is accomplished by means of both targeted regulation and stochastic changes, that likely play distinct functional roles in the stabilization and establishment of the newly formed allohexaploid plants as competitive populations and new species [12,15,16].

## Methods

### Plant lines

Two newly synthesized allohexaploid wheat lines with the same genome combination but different genotypes of one parental species (tetraploid wheat) and an identical genotype of the other parental species (a diploid goat-grass), designated as Allo-AT5 and Allo-AT9, were used. These two lines were produced by crossing *Triticum turgidum *ssp. *durum *cv. Inbar (line TTR04) (for Allo-AT5) or *T. turgidum *ssp. *carthlicum *(Line TTH01) (for Allo-AT9) with *Aegilops tauschii*(line TQ27), followed by genome-doubling with colchicine treatment [8]. Because these two allohexaploid wheat lines shared the same genotype of the diploid goat-grass species *Aegilops tauschii *(line TQ27) as the paternal parent, while the maternal parents were two different subspecies of *T. turgidum *(*durum *and *carthlicum*), the effect of the variable tetraploid parents on global gene expression of the allohexaploid lines could be addressed. The synthetic allohexaploids, produced by Ozkan *et al*. [8], and the parental lines were obtained from the seed collection of the Weizmann Institute of Science. The lines were self-pollinated for several generations, and two successive selfed generations (S4 and S5) were used in this study. All plants were grown in controlled growth chambers at 22/20°C day/night of 12 h day length.

### Multicolor genomic *in situ *hybridization (GISH)

The protocol was essentially as described by Han *et al*. [45] with minor modifications. Specifically, genomic DNA was isolated from three putative diploid progenitors of common wheat, *T. urartu*, *Ae. speltoides *and *Ae. tauschii*. DNA of *T. urartu *and *Ae. tauschii *was labelled with Chroma Tide Alexa Fluor 488-5-dUTP (Invitrogen, Cat. No. C11397) and Texas Red-5-dCTP (Perkin Elmer, Cat. No. NEL 426(Waltham, Massachusetts, USA), respectively, by nick translation. DNA of *Ae. speltoides *was used as a blocker at a ratio of 100:1 to the probe. Slide denaturation, hybridization and washing conditions were as described by the manufacturer's protocol (Invitrogen, Cat. No. C11397). Slides were examined under an Olympus fluorescence microscope (Olympus Cooperation, China Ltd,. Beijing, China) and digitally photographed.

### Microarray

Total RNA was extracted using Trizol reagent (Invitrogen) and purified through RNeasy Mini Spin Columns (Qiagen, Shanhai, China). The integrity of RNA was determined with an Agilent Bioanalyser2100 Eukaryote Total RNA Nano Series II system (Santa Clara, California, USA). The second leaf of three-leaf-stage seedlings was the source tissue for all RNA isolations. Pooled seedlings (10 plants for each replication) were used to represent each sample, with two biological replications. RNAs of the parental lines (*T. turgidum *and *Ae. tauschii*) were mixed at a ratio of 2:1 to generate the empirical MPVs for each of the synthetic allohexaploid lines. The microarray transcriptional profiling was performed by the Affymetrix, Inc. at the Gene Company Ltd. (Shanghai, China), as described in the GeneChip^® ^Expression Analysis Technical Manual. The microarrays are being submitted to The National Centre for Biotechnology Information's Omnibus Repository, and are available under the accession number GSE29882.

### Data normalization and analysis

The raw *CEL *data were normalized with the Robust Multichip Average (RMA) method [65] using the *R *software 'limma' package [66] To identify differentially expressed genes, we used an empirical Bayesian-based method [67] to construct statistics and compute the relative *P*-values [66]. The traditional *t- *test is a gene-by-gene method, the relative statistics (*t*-statistics) is a ratio of mean and standard deviation which could be seriously influenced by any unexpected noise. The new empirical Bayesian method overcomes this weakness by providing new variance estimation. Instead of using data from each single gene, the new eBayesian method proposes utilizing the information from the whole data set. Final variance estimation is a weighted average between traditional variance estimation value and the whole data set variance estimation. This moderated *t*-statistics has very robust property for small numbers of arrays and allows for incomplete data arising from spot-filtering or spot-quality weights. Genes that were differentially expressed among genotypes were identified by a cut-off *P-*value < 0.05. The present (P) or absent (A) calls of each probe-set were carried out by the MAS5 method using GCOS (Affymetrix Technologies, The Affymetrix, INC. Santa Clara, CA, USA) with default parameters. The differently expressed genes which did not show both present calls (P) in the two biological replications in at least one of the genotypes of each comparison were excluded from further analysis [68], and 29,650 genes were identified as expressed in our plant lines.

### Real-time quantitative (q) RT-PCR

To confirm the non-additive expression obtained from microarray data, we performed real-time (q) RT-PCR analysis of nine selected genes (see Additional file 7). Three independent batches of RNA were isolated as biological replications. Four house-keeping genes, *Gadph *(GenBank accession: EU022331.1), *Tubulin *(GenBank accession: U76558.1), *Actin *(GenBank accession: BG904635.1) and *Elf *(GenBank accession: AK334915.1) which are known to be constitutively expressed in wheat [69,70], were used to normalize the (q) RT-PCR data.

### Gene ontology

Each of the two groups of genes, that is, showing parental dominance expression and non-additive expression, both in their entity and as further categorized subgroups, were analyzed with Gene Ontology (GO) annotation using AgriGO http://bioinfo.cau.edu.cn/agriGO/index.php, a web-based database tool for gene ontology annotations of agricultural crops(Bioinformatics Center, China Agricultural University, Beijing, China) We used the Singular Enrichment Analysis (SEA) tool (**Bioinformatics Center, China Agricultural University, Beijing, China) **to perform the GO annotations and statistical analysis for GO term-enrichment. The SEA analysis computed GO term enrichment in one set of genes by comparing it to another set, then named the target and reference list, respectively [71]. The Fisher' exact test was used for statistical analysis with Hochberg FDR-based multi-test (*P-*value < 0.05).

## Abbreviations

GISH: genomic in situ hybridization; GO: Gene Ontology; MPVs: mid-parent values.

## Competing interests

The authors declare that they have no competing interests.

## Authors' contributions

BL conceived and designed the research. BQ, WH, XZ and JG performed the array data analysis and q-RT-PCR experiments. BZ, NZ, CM, HZ and FH took care of the plants and performed the cytological analysis. JP, BQ, FH and BL wrote the manuscript.

## Supplementary Material

Additional file 1 **Additional Table 1**. Transcriptomic divergence between the tetraploid and diploid parental species, *Triticum turgidum *and *Aegilops tauschii*. Click here for file

Additional file 2 **Additional Figure 1**. Venn diagrams illustrating the number and proportion (%) of differentially expressed genes between the parental species for each of the allohexaploid wheat lines (Allo-AT5 and Allo-AT9) that are common between the two parental species pairs (TTR04 vs. TQ27 and TTH01 vs. TQ27, respectively). Based on statistically tested differential expression. Click here for file

Additional file 3 **Additional Table 2**. Differentially expressed genes between each of the wheat allohexaploid lines and their parental species. Click here for file

Additional file 4 **Additional Table 3**. Number and percentages of parent expression bias/dominance genes in MPVs and each synthetic line. Click here for file

Additional file 5 **Additional Table 4**. Gene Ontology annotation of the 147 probe set IDs (genes) that showed consistent parental dominance expression (Group III) in both nascent allohexaploid wheat lines (Allo-AT5 and Allo-AT9) at both generations (S4 and S5). Click here for file

Additional file 6 **Additional Table 5**. Gene Ontology annotation of the 27 probe set IDs (genes) that showed consistent non-additive expression (Group III) in both nascent allohexaploid wheat lines (Allo-AT5 and Allo-AT9) at both generations (S4 and S5). Click here for file

Additional file 7 **Additional Table 6**. Validation of the microarray data by (q)-RT-PCR. Click here for file
