# Supplementary material for: Global transgenerational gene expression dynamics in two newly synthesized allohexaploid wheat (Triticum aestivum) lines
Source: BMC Biol. 2012 Jan 26;10:3. doi: 10.1186/1741-7007-10-3 (PMC3313882; doi:10.1186/1741-7007-10-3)
Supplement: Additional file 1 — Additional Table 1. Transcriptomic divergence between the tetraploid and diploid parental species, Triticum turgidum and Aegilops tauschii. [file 1741-7007-10-3-S1.DOC]

**Additional file 1 Transcriptomic divergence between the tetraploid and diploid parental species, *Triticum turgidum* and *Aegilops tauschii***

| Comparison between parental species* | *T.turgidum* ssp. *durum*, cv TTR04) *vs. Ae.tauschii*, TQ27 | *T.turgidum* ssp. *durum*, cv TTH01) *vs. Ae.tauschii*, TQ27 | *T.turgidum* ssp. *durum*, cv TTR04 *vs.*cv TTH01 |
| --- | --- | --- | --- |
| Total no. and (%^a^) of differentially expressed genes | 10,408  (35.1) | 12,850  (43.3) | 2459  （8.3） |
| No. and (%^b^) of  up-regulated genes | 5,091  (48.9) | 6,871  (53.5) | 874  （35.5） |
| No. and (%^b^) of  down-regulated genes | 5,317  (51.1) | 5,979  (46.5) | 1585  （64.5） |
| No. and (%^b^) of genes only showing present calls (P) in the tetraploid parent | 3,675  (35.3) | 4,310  (33.5) | --- |
| No. and (%^b^) of genes only showed present calls (P) in the diploid parent | 2,571  (24.7) | 2,231  (17.4) | --- |
| Total no. and (%^b^) of genes showing present calls (P) | 6,246  (60.0) | 6,541  (50.9) | --- |

*difference in expression level p<0.05 (see Methods)

^a^of all expressed genes.

^b^of differentially expressed genes.
