# Supplementary figures and images for: Global transgenerational gene expression dynamics in two newly synthesized allohexaploid wheat (Triticum aestivum) lines
Source: BMC Biol. 2012 Jan 26;10:3. doi: 10.1186/1741-7007-10-3 (PMC3313882; doi:10.1186/1741-7007-10-3)

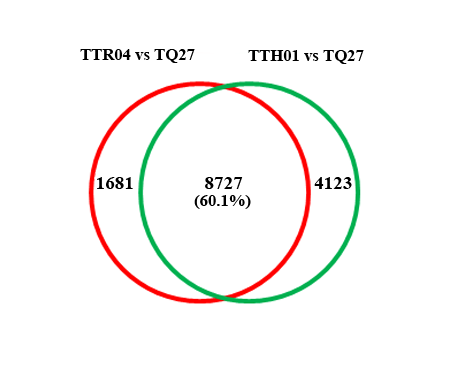

Supplement: Additional file 2 — Additional Figure 1. Venn diagrams illustrating the number and proportion (%) of differentially expressed genes between the parental species for each of the allohexaploid wheat lines (Allo-AT5 and Allo-AT9) that are common between the two parental species pairs (TTR04 vs. TQ27 and TTH01 vs. TQ27, respectively). Based on statistically tested differential expression. [file 1741-7007-10-3-S2.TIFF]
