# Supplementary material for: Global transgenerational gene expression dynamics in two newly synthesized allohexaploid wheat (Triticum aestivum) lines
Source: BMC Biol. 2012 Jan 26;10:3. doi: 10.1186/1741-7007-10-3 (PMC3313882; doi:10.1186/1741-7007-10-3)
Supplement: Additional file 3 — Additional Table 2. Differentially expressed genes between each of the wheat allohexaploid lines and their parental species. [file 1741-7007-10-3-S3.DOC]

**Additional file 3** Differentially expressed genes between each of the synthetic wheat allohexaploid lines and their parental species.

| Synthetic line and generation | Allo-AT5 | | Allo-AT9 | |
| --- | --- | --- | --- | --- |
|  | S4 | S5 | S4 | S5 |
| No. and (%^a^) of differentially expressed genes between a given allohexapolyploid line and their tetraploid parental line *T.turgidum*, at different selfed generations (S) | 2795  (9.4) | 3993  (13.5) | 4412  (14.9) | 4216  (14.2) |
| No. and (%^a^) of differentially expressed genes between a given allohexapolyploid lines and their diploid parental line, *Ae.tauschii*, at different selfed generations (S) | 9777  (33.0) | 13074  (44.1) | 10812  (36.5) | 9700  (32.7) |

^a^of all expressed genes.
