# Supplementary material for: Global transgenerational gene expression dynamics in two newly synthesized allohexaploid wheat (Triticum aestivum) lines
Source: BMC Biol. 2012 Jan 26;10:3. doi: 10.1186/1741-7007-10-3 (PMC3313882; doi:10.1186/1741-7007-10-3)
Supplement: Additional file 4 — Additional Table 3. Number and percentages of parent expression bias/dominance genes in MPVs and each synthetic line. [file 1741-7007-10-3-S4.DOC]

**Additional file 4** **Number and percentage of parent expression bias/dominance genes in MPVs and each synthetic line.**

| Synthetic lines | Allo-AT5 | | | | | Allo-AT9 | | | | |
| --- | --- | --- | --- | --- | --- | --- | --- | --- | --- | --- |
|  | AT5S4  Expression biased genes | AT5S5  Expression biased genes | MPV5  Expression biased genes | AT5S4 Expression dominance genes | AT5S5 Expression dominance genes | AT9S4 Expression biased genes | AT9S5 Expression biased genes | MPV9 Expression biased genes | AT9S4 Expression dominance genes | AT9S5 Expression dominance genes |
| No. and (%^c^) of high diploid expression biased or dominance genes | 453  (61.9) | 359  (56.3) | 549  (72.5) | 279  （54.2） | 233  （50.7） | 523  (46.2) | 484  (42.3) | 220  (39.5) | 387  （44.4） | 363  （40.3） |
| No. and (%^c^) of low diploid expression biased or dominance genes | 279  (38.1) | 279  (43.7) | 208  (27.5) | 236  （45.8） | 227  （49.3） | 608  (53.8) | 660  (57.7) | 337  (60.5) | 484  （55.6） | 537  （59.7） |
| No. and (%^d^) of total diploid expression biased or dominance genes | 732  (7.0) | 638  (6.1) | 757  (7.3) | 515  （4.9） | 460  （4.4） | 1131  (8.8) | 1144  (8.9) | 557  (4.3) | 871  （6.8） | 900  （7.0） |
| No. and (%^e^) of high tetraploid expression biased or dominance genes | 3563  (59.5) | 3628  (56.7) | 2979  (60.4) | 1192  （53.0） | 1355  （51.8） | 3829  (56.7) | 3389  (55.3) | 4166  (57.5) | 973  （58.9） | 823  （54.3） |
| No. and (%^e^) of low tetraploid expression biased or dominance genes | 2426  (40.5) | 2771  (43.3) | 1954  (39.6) | 1058  （47.0） | 1260  （48.2） | 2923  (43.3) | 2743  (44.7) | 3078  (42.5) | 679  （41.1） | 692  （45.7） |
| No. and (%^d^) of total tetraploid expression biased or dominance genes | 5989  (57.5) | 6399  (61.5) | 4933  (47.4) | 2250  （21.6） | 2615  （25.1） | 6752  (52.5) | 6132  (47.7) | 7244  (56.4) | 1652  （12.9） | 1515  （11.8) |

F represented the synthetic allohexaploid lines; P, paternal diploid lines; and M, maternal tetraploid lines. High-paternal expression bias/dominance, F =P, F>M, P>M; high-maternal expression bias/dominance, F =M, F>P, M>P; low-paternal expression bias/dominance, F =P, F<M, P<M; low-maternal expression bias/dominance, F =M, F<P, M<P. ^c^of all genes that showed diploid expression dominance. ^d^of all genes differ between parent lines. ^e^of all genes that showed tetraploid expression dominance.
