# Supplementary material for: Global transgenerational gene expression dynamics in two newly synthesized allohexaploid wheat (Triticum aestivum) lines
Source: BMC Biol. 2012 Jan 26;10:3. doi: 10.1186/1741-7007-10-3 (PMC3313882; doi:10.1186/1741-7007-10-3)
Supplement: Additional file 7 — Additional Table 6. Validation of the microarray data by (q)-RT-PCR. [file 1741-7007-10-3-S7.DOC]

**Additional file 7 Validation of the microarray data by (q)-RT-PCR**

| Probe set ID | Putative function | Nonadditivity | FCM | FCR |
| --- | --- | --- | --- | --- |
| Ta.12195.1.A1_at | Transcribed sequence | Allo-AT5 at S4 | 4.8 | 21.3** |
|  |  | Allo-AT5 at S5 | 3.1 | 20.2** |
|  |  | Allo-AT9at S5 | 7.2 | 1.3* |
| TaAffx.57297.1.S1_at | mitochondrial matR gene & nad1 gene | Allo-AT5 at S4 | 0.04 | 0.6** |
|  |  | Allo-AT5 at S5 | 0.04 | 0.3** |
|  |  | Allo-AT9at S4 | 0.1 | 0.4** |
|  |  | Allo-AT9at S5 | 0.1 | 0.2** |
| Ta.3976.2.S1_x_at | Transcribed sequence with weak similarity to protein ref:NP_197841.1 (A.thaliana) flavanone 3-hydroxylase-like protein (*Arabidopsis thaliana*) | Allo-AT5 at S4 | 0.6 | 0.8** |
| TaAffx.28462.1.S1_at | Transcribed sequence | Allo-AT5 at S4 | 7.7 | 49.4** |
|  |  | Allo-AT5 at S5 | 9.8 | 88.9** |
|  |  | Allo-AT9at S4 | 18.5 | 116.1** |
|  |  | Allo-AT9at S5 | 12.3 | 131.7** |
| Ta.10807.1.A1_at | Transcribed sequence | Allo-AT5 at S4 | 0.5 | 1.3** |
|  |  | Allo-AT5 at S5 | 0.4 | 0.7** |
|  |  | Allo-AT9at S4 | 0.5 | 0.4** |
|  |  | Allo-AT9at S5 | 0.5 | 0.3** |
| TaAffx.128796.1.S1_at | Transcribed sequence | Allo-AT5 at s4 | 0.4 | 1.3** |
|  |  | Allo-AT5 at s5 | 0.4 | 0.5** |
|  |  | Allo-AT9at S4 | 0.6 | 0.5** |
|  |  | Allo-AT9at S5 | 0.6 | 0.4* |
| Ta.21601.1.A1_at | Transcribed sequence | Allo-AT9at S4 | 2.0 | 0.02** |
|  |  | Allo-AT9at S5 | 1.7 | 10.9** |
| Ta.30798.3.S1_at | vacuolar-processing enzyme precursor, putative, expressed, Expect:0, match=501/376 | Allo-AT9at S4 | 0.4 | 1.2* |
|  |  | Allo-AT9at S5 | 0.4 | 0.5** |
| Ta.5385.1.S1_at | peroxidase | Allo-AT5 at S4 | 0.3 | 0.2** |
|  |  | Allo-AT5 at S5 | 0.1 | 0.1** |
|  |  | Allo-AT9at S4 | 0.3 | 0.1** |
|  |  | Allo-AT9at S5 | 0.3 | 0.1** |

^†^Nonadditivity refers to expression based on the microarray data in one or both of the synthetic allohexaploid wheat lines (Allo-AT5 and Allo-AT9) at one or both of the selfed generations (S4 and S5);

FCM: fold change detected by microarray

FCR: fold change detected by (q)-RT-PCR

* p value≤0.05; ** p value≤0.01.
